# Mechanism of Silica Nanoparticle-Induced Particulate Fouling in Vacuum Membrane Distillation

**DOI:** 10.3390/membranes14040076

**Published:** 2024-03-27

**Authors:** Yejin Lee, Suyoung Jeong, Jae-Hyuk Kim, Sanghyun Jeong

**Affiliations:** School of Civil and Environmental Engineering, Pusan National University, Busan 46241, Republic of Korea; lyj990102@pusan.ac.kr (Y.L.); jaehyuk.kim@pusan.ac.kr (J.-H.K.)

**Keywords:** membrane distillation, vacuum membrane distillation, membrane fouling

## Abstract

Membrane distillation (MD) is a process driven by the vapor pressure difference dependent on temperature variation, utilizing a hydrophobic porous membrane. MD operates at low pressure and temperature, exhibiting resilience to osmotic pressure. However, a challenge arises as the membrane performance diminishes due to temperature polarization (TP) occurring on the membrane surface. The vacuum MD process leverages the application of a vacuum to generate a higher vapor pressure difference, enhancing the flux and mitigating TP issues. Nevertheless, membrane fouling leads to decreased performance, causing membrane wetting and reducing the ion removal efficiency. This study investigates membrane fouling phenomena induced by various silica nanoparticle sizes (400, 900, and 1300 nm). The patterns of membrane fouling, as indicated by the flux reduction, vary depending on the particle size. Distinct MD performances are observed with changes in the feed water temperature and flow rate. When examining the membrane fouling mechanism for particles with a porosity resembling actual particulate materials, a fouling form similar to the solid type is noted. Therefore, this study elucidates the impact of particulate matter on membrane fouling under diverse conditions.

## 1. Introduction

A significant challenge in many parts of the world is the shortage of natural freshwater resources, affecting approximately one billion people who lack access to clean water [1]. Developing technology for producing clean water is crucial to addressing this issue. Despite the abundance of water on Earth, with 97% being seawater and an additional 2% locked in glaciers, only a small fraction (less than 0.5%) is available as freshwater [2]. Given the predominance of seawater, the development of desalination technologies becomes paramount. In contrast to conventional desalination processes, such as multi-stage flash (MSF), multiple-effect distillation (MED), reverse osmosis (RO), or electrodialysis (ED) [3,4], the membrane distillation (MD) process is a thermal membrane separation method. It utilizes a hydrophobic microporous membrane to separate feed brine from permeate water, based on vapor pressure differences resulting from the temperature difference. The hydrophobic nature of the membrane only allows vapor to pass through, preventing the passage of liquid. Consequently, vapor moves from the hot solution side with higher vapor pressure to the cold distillate side with lower vapor pressure [4,5,6]. The MD process offers several advantages, including (1) a lower operating temperature, (2) reduced energy costs, and (3) compact facility design, which contribute to the growing interest in MD [5].

MD processes are utilized not only in water treatment but also in various applications, such as air purification, humidification, and dehumidification [7,8,9,10]. In the process of dehumidifying and purifying air using a liquid desiccant, particulate matter in the air can be collected and, during the subsequent re-concentration of the diluted liquid desiccant using MD, these particles may accumulate on the membrane surface [11]. Consequently, the accumulation of particulate matter leads to membrane fouling, a significant issue arising from the stacking of organic and inorganic matter on the membrane surface, thereby obstructing the membrane pores and causing a reduction in the MD process efficiency and flux [12,13]. During the MD process, membrane fouling diminishes the membrane hydrophobicity as particles clog the membrane, ultimately resulting in membrane pore wetting [14,15]. Therefore, the precise characterization of membrane fouling is essential for establishing effective control measures.

To assess the impact of particulate material on the MD process, silica nanoparticles (SNPs) were employed due to their universal presence as inorganic contaminants on the surface of the membrane’s active layer. SNPs exhibit low reactivity with water, facilitating the observation of particulate fouling mechanisms [14]. While recent studies have explored the influence of solid silica on the MD process, they may not provide accurate membrane fouling information, as most particulate matter exhibits a porous structure rather than a solid type [16,17,18].

The MD process encompasses various types, with direct contact MD (DCMD) being prevalent in more than 60% of MD studies, due to its simple operation and the occurrence of condensation inside the membrane module [19,20,21]. However, DCMD has drawbacks, such as heat conduction through the membrane due to direct contact of the feed water with the cool permeate side. This can result in TP on the brine side, leading to a decline in the permeate flux [21]. However, the vacuum MD (VMD) process is a cost-effective membrane separation technology, operated by a vacuum pump on the permeate side to extract vapor and create a higher vapor pressure difference [22,23,24]. Additionally, the flux decline caused by TP is minimized during the VMD process, as the vapor pressure on the permeate side is determined by decompression, without the need for a coolant. Unlike the DCMD process, the VMD process can prevent feed water contamination, as it does not involve direct contact with the permeate side [21].

In this study, we examined the effect of the SNP size, feed temperature, flow rate, and SNP type within a lab-scale VMD system. We utilized a particle size analyzer (PSA) and transmission electron microscopy (TEM) to observe the size and shape of the SNPs. Additionally, scanning electron microscopy (SEM) and water contact angle (CA) measurements were employed to characterize the extent of membrane fouling.

## 2. Materials and Methods

### 2.1. Materials

To fabricate the SNPs, ammonia solution (28%, Junsei Chemical, Tokyo, Japan), ethyl alcohol (99.9%, Samchun Chemicals, Seoul, Republic of Korea), and ethyl silicate (98.5%, EP) (or tetraethyl orthosilicate, TEOS, Daejung Chemicals & Metals, Gyeonggi-do, Republic of Korea) were used.

### 2.2. Membrane

In this experiment, a commercially available polyvinylidene fluoride (PVDF) hydrophobic membrane, with a pore size of 0.22 μm (GVHP14250, Durapore, Darmstadt, Germany), was employed. The membrane’s CA, measured using a CA meter (Phoenix-10, SEO, Gyeonggi-do, Republic of Korea), was determined to be 100 ± 5°, indicating its hydrophobic nature. The effective membrane area was 0.0019 m^2^, with dimensions of 0.0765 m in length and 0.0245 m in width. The membrane had a thickness of 122 μm and a porosity of 72%. To prevent membrane deformation due to the vacuum and maintain a flat state, a 3D-printed feed spacer was fabricated in the laboratory and utilized in the experimental setup.

### 2.3. VMD Setup

**Figure 1:**
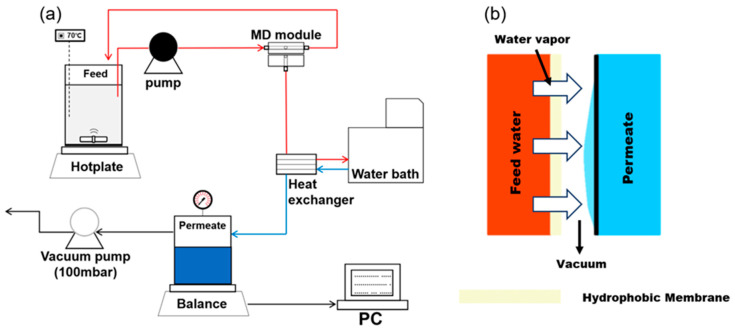
Schematic diagrams of: (**a**) the laboratory-scale VMD setup, and (**b**) the principle of the VMD process.

Figure 1 shows the schematic diagram of the lab-scale VMD setup. A gear pump was used to ensure a continuous flow of the feed solution, and two different feed flow rates (0.4 and 0.6 L/min) were tested. On the permeate side, a vacuum pump created a vacuum inside the permeate tank, maintaining a fixed vacuum pressure of 100 mbar. The feedwater was heated using a hotplate, stirring continuously at 300 rpm to uniformly disperse the SNPs. The feed temperature was varied between 60 and 80 °C. To monitor the permeate flux, a digital balance recorded the increase in the permeate weight, and the data were automatically transferred to a computer every minute. The temperature of the permeate water was cooled to 20 °C, using a water chiller circulating water through a heat exchanger.

### 2.4. Silica Nanoparticles

Silica nanoparticles (SNPs) were synthesized using the Stöber method [25]. Firstly, solution “I”, consisting of ethyl alcohol, deionized (DI) water, and ammonia solution at a ratio of 5:1:1, was stirred for 30 min. An ethanolic solution of tetraethyl orthosilicate (TEOS) (1.3 mL, 4:1) was rapidly added to 6.5 mL of solution “I” and stirred for 2 h. Subsequently, the mixture was centrifuged and washed with ethanol three times. The obtained product was dried in an oven at 110 °C for at least 5 min. Larger-sized SNPs were obtained by adjusting the ratio and repeating the experiment, using the same method. Recognizing that SNPs of different sizes might have distinct effects on membrane fouling, three different sizes of SNPs (400, 900, and 1300 nm) were prepared, and comparative experiments were conducted. To simulate real particles, the experiment was also performed with 900 nm-sized hollow-type SNPs, prepared according to a previous study [26].

### 2.5. Experimental Conditions

To investigate the effect of the feed water temperature, feed flow rate, silica particle size, and type of silica particle on membrane fouling due to SNPs, various experimental conditions were tested, as summarized in Table 1. The SNPs were dissolved in 2 L of DI water to achieve a concentration of 1 g/L. To ensure the complete dispersion of the SNPs, the experiment commenced after stirring them at 500 rpm for 24 h.

Initially, the experiments were carried out with various feed temperatures (60, 70, and 80 °C) (E1–E3). To examine the influence of the SNP particle size on membrane fouling, SNP sizes ranging from 400 to 1300 nm were employed in different experiments (E2, E4, E5). Additionally, experiments with varied flow rates were conducted to investigate the impact on the accumulation mechanism of silica particles on the membrane (E2, E6). In the preceding experiments (E1–E6), only solid-type silica was utilized. However, recognizing that actual particulate matter often exhibits a hollow structure, an experiment was conducted with 900 nm hollow-type silica for comparison with the solid type (E7). In VMD, the primary driving force stems from the difference in vapor pressure based on the temperature disparity, with vacuum pressure serving as an additional driving force. Through preliminary experiments and a review of the literature, we determined that the effect of vacuum pressure would not be discussed in this paper.

As water on the feed side is condensed through the membrane and heat exchanger, the water volume on the permeate tank side increases, which was automatically recorded on the computer every minute. The experiment continued until the volume concentration factor (*VCF*) reached 2.5 or 4.0, calculated using Equation (1):

(1) $$VCF=\frac{V_{i}}{V_{i}-V_{p}}$$

where *V_i_* represents the initial volume of the feed tank, and *V_p_* is the permeate volume. Additionally, the permeate flux (L/m^2^h, LMH) was calculated using Equation (2):

(2) $$J=\frac{∆m}{∆t\times A}$$

where Δ*m* is the weight increase in the permeate tank, Δ*t* is the experiment time, and *A* is the effective membrane surface area.

To compare the performance under different operating conditions, the fouling ratio was calculated using Equation (3) [14]:

(3) $$Fouling\;ratio=\frac{J}{J_{0}}$$

where *J*_0_ is the initial permeate flux, and *J* is the final permeate flux.

Following the experiments, cleaning was carried out with DI water, by circulating it at 1.0 L/min for 5 h to clean the equipment.

### 2.6. Analytical Methods

After the experiment, the membrane was observed after drying in an oven at 110 °C for 1 d. PSA (Beckman Coulter, CA, USA) and TEM (HITACHI, Tokyo, Japan) were used to observe the size and shape of the prepared SNPs, respectively. To measure the TEM, the TEM grid was coated with the sample 5 times. The membrane surface was observed using SEM (SUPRA, Zeiss, Oberkochen, Germany) at a magnification of 5000×. The hydrophobicity of the membrane surface was investigated using a CA meter (Phoenix-10, SEO, Gyeonggi-do, Republic of Korea).

## 3. Results

### 3.1. Preparation of the SNPs

**Figure 2:**
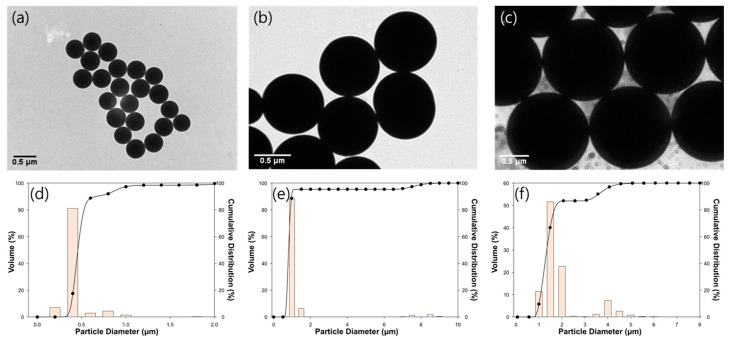
TEM images and graphs of particle size distribution of SNPs measured using PSA: (**a**,**d**) 400 nm, (**b**,**e**) 900 nm, and (**c**,**f**) 1300 nm.

Figure 2 shows the shape of the SNPs observed using TEM. Spherical solid-type SNPs with high uniformity were observed, indicating that the SNPs were well made in a spherical shape, which can minimize the effect of the shape in regard to membrane particulate matter fouling (Figure 2a–c) [27]. According to the PSA results (Figure 2d–f), it was confirmed that the SNPs were made according to the targeted size, with mode values of 431 nm, 910 nm, and 1321 nm, respectively.

### 3.2. Factors on Particulate Fouling on MD Membrane

#### 3.2.1. Effect of Feed Water Temperature

**Figure 3:**
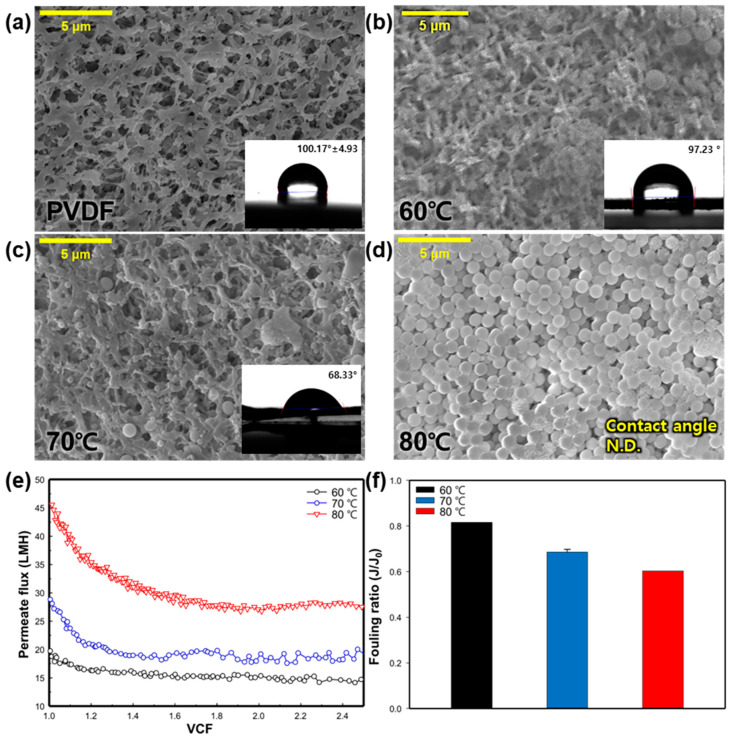
Effect of feed temperature on silica particulate membrane fouling; SEM images (5000×) and water CA value of membrane surface: (**a**) clean (pristine) membrane, (**b**) 60 °C, (**c**) 70 °C, and (**d**) 80 °C. Effect of feed temperature on: (**e**) change of flux in regard to the function of the VCF, and (**f**) the fouling ratio (*J*/*J*_0_).

To examine the impact of the feed water temperature, the experiment was conducted under three different temperature conditions (60, 70, and 80 °C), with a constant permeate temperature of 20 °C. Figure 3a–d shows the SEM images and CA of the virgin and silica-fouled membranes at various feed temperatures. In all cases involving the silica-fouled membranes, the accumulation of SNPs on the membrane surface was evident. When inspecting the membrane after conducting the experiment at 60 °C, the least amount of SNPs was observed adhering to the membrane surface. The SEM image of the membrane surface at 70 °C showed a larger amount of SNPs compared to 60 °C, while at 80 °C, SNPs covered the membrane surface, making it challenging to make observations. At 80 °C, it was not possible to measure the contact angle (CA) due to the hydrophilic characteristics of the SNPs. The CA at 60 °C and 70 °C was 97.23° and 68.33°, respectively; whereas, the pristine membrane before the MD process exhibited a CA of 100.17 ± 4.93°.

Figure 3e illustrates the change in the permeate flux with varying feed water temperatures. At a feed water temperature of 60 °C, the initial permeate flux was 19.75 LMH. The flux remained relatively constant without a significant decrease until a VCF of 2.5, as evident in the fouling ratio of 0.82 (Figure 3f). When the feed water temperature was set at 70 °C, the initial permeate flux was 28.8 LMH. However, it rapidly decreased after a VCF of 1.2, maintaining a relatively constant level until the end of the experiment. Under a feed water temperature of 80 °C, the initial permeate flux was the highest at 45.4 LMH. Despite the increased temperature leading to a higher vapor pressure difference and permeate production [28], the permeate flux at 80 °C also decreased sharply after a VCF of 1.2, showing a similar pattern to the 70 °C condition. The fouling ratio at 70 °C and 80 °C was 0.68 and 0.60, respectively (Figure 3f). The observed flux reduction is attributed to the accumulation of SNPs on the membrane. As SNPs accumulate on the membrane, they block the membrane surface and pores, resulting in a decrease in the flux. Additionally, the hydrophobicity of the membrane surface is completely lost at higher feed temperatures (as seen in Figure 3b,d), leading to a further reduction in the liquid entry pressure (LEP) of the membrane. A decreased LEP makes it easier for the feed solution to pass through the membrane pores, thereby increasing the likelihood of membrane fouling [29]. At 80 °C, there is a risk of rapid membrane damage due to an increase in the LEP and rapid vapor permeation, causing contaminants to quickly attach to the membrane. This is evident in the high amount of SNPs found on the membrane surface (Figure 3b). In addition, the 60 °C condition was deemed unsuitable as it took a long time to observe membrane fouling due to the low and constant permeate flux. Therefore, the temperature for the other experiments was fixed at 70 °C.

#### 3.2.2. Effect of SNP Size

**Figure 4:**
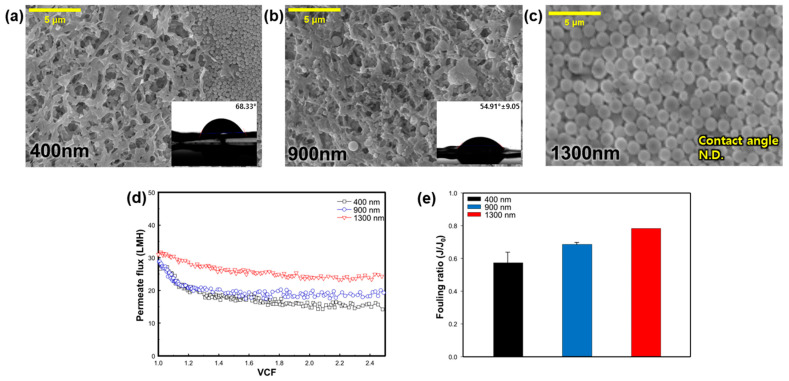
Effect of SNP size on silica particulate membrane fouling; SEM images (5000×) and CA of membrane surface: (**a**) 400 nm, (**b**) 900 nm, and (**c**) 1300 nm. Effect of silica nanoparticle size on: (**d**) change of flux in regard to the function of the VCF, and (**e**) the fouling ratio (*J*/*J*_0_).

To investigate the impact of the silica particle size, experiments were conducted using three different-sized SNPs—400 nm, 900 nm, and 1300 nm. Based on the previous experiment, the temperature of the feed and permeate were fixed at 70 and 20 °C, respectively. Figure 4a–c shows the SEM images and CA values after the MD operation. In Figure 4b,d, it is evident that SNPs accumulated on the membrane surface in all cases. Observing the membrane after the experiment with the 400 nm-sized SNPs revealed an accumulated group of SNPs on the membrane surface. In the condition involving 900 nm-sized SNPs, larger particles were observed on the membrane surface, while with the 1300 nm condition, SNPs covered the membrane surface, making it difficult to observe the membrane. The CA value of the pristine membrane before the MD process was 100.17°, but it decreased to 68.33° and 54.91° with 400 nm and 900 nm SNPs, respectively (Figure 4a,b). In Figure 4c, the membrane surface could not be observed due to its complete coverage by SNPs, so the CA could not be measured. As expected, larger SNPs adhered more easily to the membrane surface, resulting in a cake layer on the surface [30]. In denser and thicker membrane fouling layers, the evaporation temperature can be lower, thereby reducing the MD performance.

Figure 4d shows the permeate flux pattern over time, according to the size of the SNPs. When the size of SNPs was 400 nm, the initial permeate flux was 28.67 LMH, but it decreased rapidly after a VCF of 1.2 and stabilized. During the 900 nm condition, the initial permeate flux was 28.83 LMH, and the flux also decreased until the VCF was 1.2, as the SNPs accumulated on the membrane surface. Under the 1300 nm condition, the initial permeate flux was 31.04 LMH, and the permeate flux decreased slowly compared to other SNPs conditions. In Figure 4e, the fouling ratio of the 400 nm, 900 nm, and 1300 nm-sized SNPs was 0.57, 0.69, and 0.78, respectively. It was confirmed that the smaller the SNP size, the greater the flux reduction rate. Comparing the SEM and CA results in Figure 4a–c, larger SNP sizes resulted in a greater amount of SNPs remaining on the membrane, making it difficult to observe the membrane surface, and the CA value also decreased. However, this could be easily removed with the fluid flow, suggesting it may not be a loss of functionality of the membrane itself, but rather a decrease in the CA value due to the hydrophilic characteristics of the SNPs. This is because SNPs with a size similar to the membrane pore size seem to block the pores in the membrane [31].

#### 3.2.3. Effect of the Flow Rate

**Figure 5:**
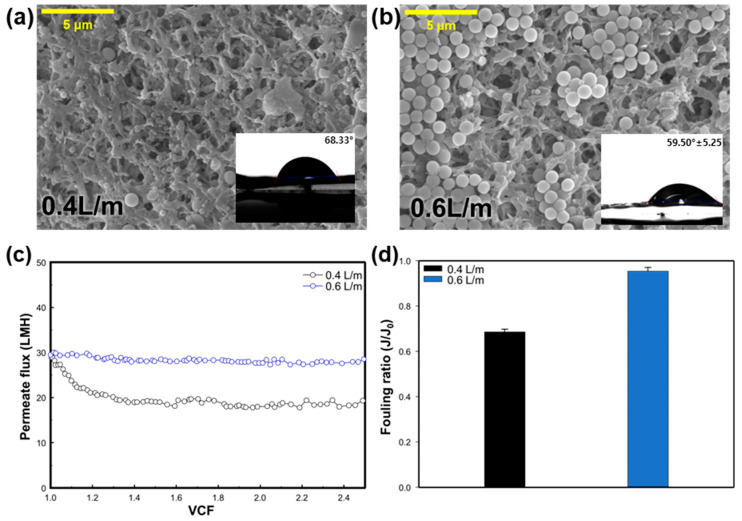
Effect of flow rate on silica particulate membrane fouling; SEM images (5000×) and CA of membrane surface: (**a**) 0.4 L/m, and (**b**) 0.6 L/m. Effect of flow rate on: (**c**) change in the flux in regard to the function of the VCF, and (**d**) the fouling ratio (*J*/*J*_0_).

In order to examine the effect of the feed flow rate on membrane fouling, experiments were conducted with a feed temperature of 70 °C, a permeate temperature of 20 °C, and an SNP size of 900 nm. Figure 5a,b depicts the SEM images and CA values of the clean membrane and silica-fouled membranes. In the SEM images, more SNPs were observed on the membrane surface at the 0.6 L/min flow rate compared to the 0.4 L/min condition. Consequently, as the flow rate increased, a higher accumulation of silica particles on the membrane was observed, and the CA was measured at 59.5 ± 5.0° (Figure 5b), indicating a greater loss of hydrophobicity compared to the membrane tested at the lower flow rate of 0.4 L/min (Figure 5a). Figure 5c shows the change in the permeate flux in regard to the function of the VCF, according to the feed water flow rate. In the condition of 0.4 L/m, the initial permeate flux was 28.83 LMH, but the flux decreased sharply until a VCF of 1.2, and remained stable thereafter. In the 0.6 L/min condition, the initial permeate flux was 29.47 LMH. The permeate flux remained stable, unlike the 0.4 L/min condition, resulting in a high fouling ratio with a value of 0.95, whereas the fouling ratio for the 0.4 L/min condition was 0.69 (Figure 5d). The Reynolds number (Re) for the 0.4 L/min and 0.6 L/min condition was 4515 and 7960, respectively, indicating turbulent flow (Re > 4000). As the turbulence increases, the fouling rate tends to decline with the rise in turbulence kinetic energy [32]. Despite the loss of hydrophobicity indicated by the CA value at 0.6 L/min, the fouling ratio suggested that the flow rate was more constant compared to the 0.4 L/min condition. Due to the faster flow rate, SNPs appear to pass through without accumulating on the membrane and are removed by the feed flow. Therefore, it was confirmed that the slower the flow rate, the more significant the membrane fouling. However, a higher flow rate requires more energy due to the elevated Reynolds number [33]. Hence, there is an appropriate flow rate that considers the energy efficiency of the system.

#### 3.2.4. Effect of SNP Type

**Figure 6:**
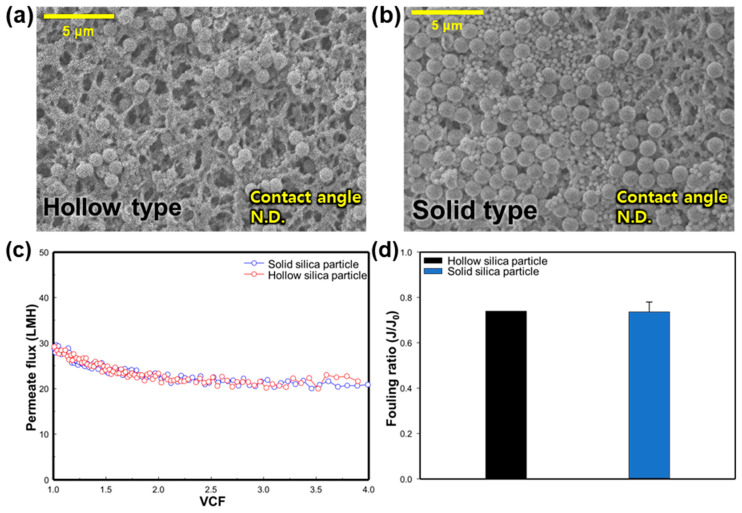
Effect of SNP type on silica particulate membrane fouling; SEM images (5000×) and CA of membrane surface: (**a**) hollow-type silica particles, and (**b**) solid-type silica particles. Effect of silica nanoparticle type on: (**c**) change in the flux in regard to the function of the VCF, and (**d**) the fouling ratio (*J*/*J*_0_).

In order to consider the various internal structures that particulate matter in an environment can have, we attempted to observe the effect of membrane fouling based on the type of silica particle. The feed and permeate temperature was set at 70 °C and 20 °C, respectively, and the experiment was conducted with silica particles with a size of 900 nm. For the hollow-type silica experiment, the feed concentration was maintained at 1 g/L, similar to the previous experiment. To observe the corresponding changes, the experiment was conducted at a VCF of 4.0. The solid-type silica was also subject to experiments under the same conditions to observe the differences between the two types. Figure 6a,b shows the SEM images and CA values of a clean membrane and silica-fouled membranes with two types of silica nanoparticles (SNPs)—hollow-type silica and solid-type silica. It appears that solid-type SNPs accumulated more on the membrane surface than the hollow type. Measurement of the CA was not possible for either type due to the presence of silica particles on the membrane. In Figure 6a, broken silica particles can be observed on the membrane surface. This is likely because the interior of hollow-type SNPs is empty, making them less durable than solid-type SNPs, and they may have broken during the MD process. Figure 6c shows the change in the permeate flux according to the type of SNPs. The initial permeate flux for the hollow-type SNP condition is 29.22 LMH, and it declined until a VCF of 1.5. The initial flux for solid-type SNPs is 28.83 LMH, and the permeate flux also decreases at a VCF of 1.5, maintaining this state thereafter, similar to the hollow type. As can be seen in Figure 6d, the fouling ratio was confirmed to be similar for both the hollow type and the solid type, at 0.74 and 0.74, respectively. This seems to be a very small effect from fouling due to the internal structure of the SNP in the range of 900 nm.

### 3.3. Possible Mechanism and Fouling Control Strategy

**Figure 7:**
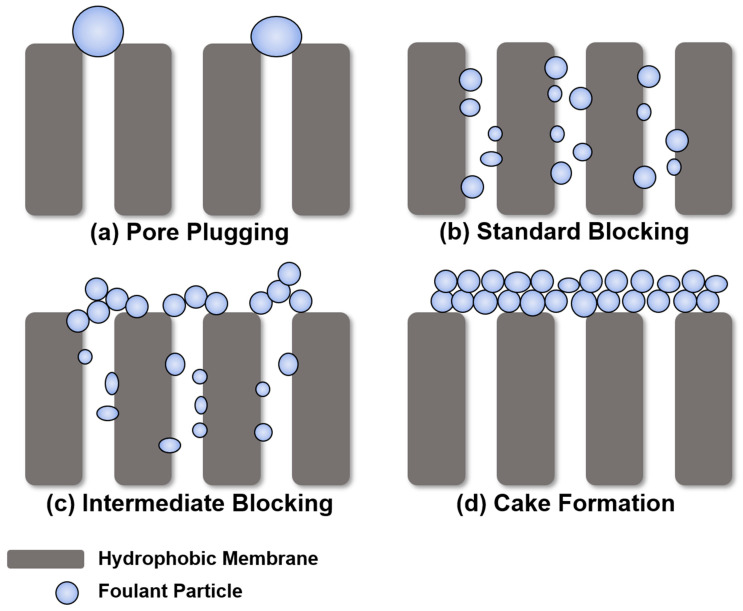
Different particulate membrane fouling mechanisms.

There are four types of membrane fouling models: pore plugging, standard blocking, intermediate blocking, and cake formation (Figure 7). Foulants that are similar in size or larger than the membrane pores can be deposited on the membrane surface, causing pore plugging (Figure 7a). If the foulant size is smaller than the pore size, it enters the pores and attaches to the inner pore surface (Figure 7b). Intermediate blocking occurs when some foulants accumulate inside the pores, and others accumulate on other deposited foulants (Figure 7c). When a uniform foulant layer is formed on the membrane surface, cake filtration occurs (Figure 7d) [34,35,36,37]. It seems that despite efforts to explore the unique factors driving membrane fouling in MD, the results did not significantly differ from those observed in typical pressure membrane systems like microfiltration (MF). Although MD operates differently and has distinct mechanisms compared to pressure membranes, the phenomenon of particulate membrane fouling appears to show similar patterns to MF due to the rejection of particulate substances by similar pore-sized membranes.

In this study, membrane fouling was observed, depending on the temperature of the feed. The higher the temperature, the more severe the membrane fouling. As the temperature rises and the LEP of the membrane increases, the feed can easily pass through the membrane, and the fouling of the membrane surface also increases [29]. Therefore, membrane fouling was more severe in the 80 °C feed water condition compared to the 60 and 70 °C conditions. There is a need to determine the appropriate feed water temperature by considering both the water flux and the membrane fouling ratio.

As a result of observing membrane fouling according to the size of the SNPs, it was observed that the smaller the particle size, the more severe the fouling. In the case of MD, similar to other membrane processes, the size of the SNP is similar to the pore size of the membrane, leading to pore clogging (similar to Figure 7a), and the water flux decreases rapidly. However, larger SNPs, which are much larger in size than the pores, form the cake layer and are easily removed from the membrane surface [38,39]. Therefore, membrane fouling was more severe for the 400 nm-sized SNP condition compared to the 900 nm and 1300 nm conditions. For particles with diameters of 900 nm and 1300 nm, which are much larger than the membrane pore size, they uniformly accumulated to form a cake layer on the membrane surface. Although the permeate flux decreased, it was not as pronounced as observed with the 400 nm-sized particles. In this case, cake formation (Figure 7d) is considered reversible fouling, allowing for easy removal with the flow of fluid. In MD, the occurrence of standard and intermediate blocking (Figure 7b,c) are also presented, but their distinction is challenging due to the potential occurrence of partial or full wetting when these types of blocking happen. Based on this, to reduce particulate membrane fouling in MD, it is necessary to use a membrane with relatively small pores than the particulate materials, or to use pretreatment, such as coagulation, to increase the particle size when small particles are introduced.

In regard to the effect of the flow rate on particulate membrane fouling by SNPs, severe membrane fouling occurred under slow flow rate conditions. As the flow rate increased, the degree of mixing on the membrane surface increased and the mass transfer coefficient within the concentration boundary layer also improved. Therefore, fewer feed-side SNPs accumulate on the membrane surface, and back diffusion occurs toward the bulk solution [40]. In this study, membrane fouling was found to be less severe under the flow rate condition of 0.6 L/m compared to the flow rate condition of 0.4 L/m. Operating at a high flow rate increases the operating costs, but from the perspective of membrane fouling, appropriate flow rate conditions must be found that consider the characteristics of particulate matter.

In addition to SNPs, things that can cause particulate membrane contamination include scaling or inorganic fouling, such as CaSO_4_ or CaCO_3_, or metal oxides caused by oxidation, such as iron or manganese. However, these membrane foulants accumulate on the membrane surface or inside pores in the form of precipitates after a reaction, so additional steps are required. However, in general, the decisive membrane fouling mechanism is not significantly different from that of SNPs.

In addition, because the size of the particles used in this study were near 1 μm, where particulate membrane fouling occurs easily, it was possible to simulate the phenomenon that happens in real-world water treatment. In particular, in an environment where particulate matter enters the MD system, we were able to make a sufficient prediction because we observed the tendency of membrane fouling to occur according to the particle size when the substances become smaller. In addition, by comparing membrane fouling caused by porous particles, it was possible to make a comparison with a more realistic environment.

## 4. Conclusions

In order to identify the factors contributing to membrane fouling during the VMD process and enhance the ability to have control over fouling mechanisms, this study investigated various factors affecting MD fouling. Synthesized SNPs were analyzed with TEM and PSA. The membrane surface image was analyzed using SEM, and membrane hydrophobicity was measured using the CA. The following conclusions were drawn from this study:

- As the feed water temperature increases, the initial permeate flux also increases due to a higher vapor pressure difference. However, the flux decline also accelerates, and membrane fouling intensifies with higher feed water temperatures.
- During the examination of the SNP size, smaller particles were found to have a more significant impact on membrane fouling due to their similarity to the pore size of the membrane.
- Under 0.4 L/min and 0.6 L/min flow rate conditions, the Reynolds number was greater than 4000, indicating a turbulent flow. As the turbulence increases, the fouling rate decreases. Therefore, a faster flow rate impedes the SNPs from accumulating on the membrane surface.
- Regarding the SNP type, the results showed similar flux and fouling patterns for both solid and hollow-type SNPs.

Therefore, this study suggests methods and operating factors to reduce fouling when particulate membrane pollutants of 400 to 1300 nm are introduced in the VMD process. These results can be used as basic data to establish a membrane fouling control strategy.

## Figures and Tables

**Table 1:** Summary of experimental conditions.

	No.	Type of Silica (Size)	Feed Temp. (°C)	Flow Rate (L/min)
Effect of temperature	E1	Solid type (900 nm)	60	0.4
	E2		70	
	E3		80	
Effect of silica size	E4	Solid type (400 nm)	70	0.4
	E5	Solid type (1300 nm)		
Effect of flow rate	E6	Solid type (900 nm)	70	0.6
Effect of silica type	E7	Hollow type (900 nm)	70	0.4

## Data Availability

The original contributions presented in the study are included in the article, further inquiries can be directed to the corresponding author/s.
